# Differences in the Proteomic and Metabolomic Response of *Quercus suber* and *Quercus variabilis* During the Early Stages of *Phytophthora cinnamomi* Infection

**DOI:** 10.3389/fmicb.2022.894533

**Published:** 2022-06-13

**Authors:** Iñigo Saiz-Fernández, Biljana Đorđević, Pavel Kerchev, Martin Černý, Thomas Jung, Miroslav Berka, Chuen-Hsu Fu, Marília Horta Jung, Břetislav Brzobohatý

**Affiliations:** ^1^Department of Molecular Biology and Radiobiology, Faculty of AgriSciences, Phytophthora Research Centre, Mendel University in Brno, Brno, Czechia; ^2^Department of Forest Protection and Wildlife Management, Faculty of Forestry and Wood Technology, Phytophthora Research Centre, Mendel University in Brno, Brno, Czechia; ^3^Forest Protection Division, Taiwan Forestry Research Institute, Taipei, Taiwan

**Keywords:** micropropagation, plant pathogen, resistance, sugars, peroxidases, glutathione S-transferase, phenylpropanoids

## Abstract

*Phytophthora cinnamomi* Rands is a cosmopolite pathogen of woody plants which during the last couple of centuries has spread all over the world from its center of origin in Southeast Asia. In contrast to Chinese cork oak (*Quercus variabilis* Blume) forests native to Asia, which are generally healthy despite the presence of the pathogen, the populations of Cork oaks (*Quercus suber* L.) in Europe have been severely decimated by *P. cinnamomi*. The present study aims at identifying the differences in the early proteomic and metabolomic response of these two tree species that lead to their differences in susceptibility to *P. cinnamomi*. By using micropropagated clonal plants, we tried to minimize the plant-to-plant differences in the defense response that is maximized by the high intraspecific genetic variability inherent to the *Quercus* genus. The evolution on the content of *Phytophthora* proteins in the roots during the first 36 h after inoculation suggests a slower infection process in *Q. variabilis* plants. These plants displayed a significant decrease in sugars in the roots, together with a downregulation of proteins related to carbon metabolism. In the leaves, the biggest changes in proteomic profiling were observed 16 h after inoculation, and included increased abundance of peroxidases, superoxide dismutases and glutathione S-transferases in *Q. variabilis* plants, which probably contributed to decrease its susceptibility to *P. cinnamomi*.

## Introduction

Cork oak forests have a great ecological and economic relevance. They are associated with a remarkable biodiversity and constitute unique ecosystems that are recognized for their ecological value and as a source of income in an economic sector that is responsible for thousands of businesses in different levels (Silva and Catry, 2006; Kim et al., 2017). Chinese cork oak (*Quercus variabilis* Blume) is one of the most widespread tree species in eastern Asia (Wang et al., 2016). It is a deciduous tree providing raw material to produce lumber, cork, silicone, and charcoal (Zhou et al., 2010). Moreover, *Q. variabilis* is one of the most ecologically valuable tree species in China (Gao et al., 2021). It is widely used for soil and water conservation, especially for the afforestation of difficult site conditions, thus playing an important role in developing the local economy and protecting ecological balance (Wei et al., 2020; Pang et al., 2021). The evergreen cork oak (*Quercus suber* L.) is naturally distributed in the western Mediterranean basin, occurring in a wide range of geographic and climatic conditions (Aronson et al., 2009). Cork oak stands are important multipurpose ecosystems that combine environmental and socio-economic services. They have a huge impact for maintaining high levels of biodiversity (Bugalho et al., 2009), while supporting diverse agroforestry practices, and provide the raw material for the cork industry. Both *Q. suber* and *Q. variabilis* are phylogenetically related belonging to the intrageneric *Quercus* group Cerris which has a disjunct Eurasian distribution (Hubert et al., 2014; Hipp et al., 2020).

Cork oaks are susceptible to the attack of *Phytophthora cinnamomi* Rands, arguably the most cosmopolite and polyphagous pathogen of woody plants and other host species (Jung et al., 2013; Hardham and Blackman, 2018). While its center of origin has recently been demonstrated to be Southeast Asia (Shakya et al., 2021), the presence of disease symptoms typical of *P. cinnamomi* infections has been reported in Europe as early as the XIX century (Bergot et al., 2004). This pathogen has been demonstrated as main causal agent of the severe decline of *Q. suber* in Spain, Portugal and Italy (Robin et al., 1998; Camilo-Alves et al., 2013; Scanu et al., 2013; Moricca et al., 2016; Avila et al., 2017; Jung et al., 2018; Seddaiu et al., 2020; Cardillo et al., 2021). Infection of *P. cinnamomi* occurs predominantly through the roots, from where it spreads upwards to the trunk leading to the formation of bleeding collar lesions (Bergot et al., 2004; Jung et al., 2018). Extensive root losses and girdling of main roots and the collar cause trees to display crown symptoms including chlorosis, wilting and, eventually, dieback and mortality (Jung et al., 2018; Seddaiu et al., 2020). Like all forest declines, *Q. sub*er decline is caused not only by the presence of a pathogen but by a complex interaction between *P. cinnamomi* root infections and predisposing factors, in particular drought (Avila et al., 2017; Homet et al., 2019; González et al., 2020), low soil fertility and soil compaction (Moreira and Martins, 2005). In contrast, native *Q. variabilis* forests in Southeast Asia contain mostly healthy trees, despite the ubiquitous natural presence of *P. cinnamomi* (Jung et al., 2017), which suggests a lower level of susceptibility to this pathogen as a consequence of long-term host-pathogen co-evolution. This hypothesis was confirmed in a soil infestation test with *P. cinnamomi* and 1-years old plants which showed that after 6 months *Q. suber* had only 13.2 ± ad 3% healthy roots left as compared to between 78.0 ± 23.6 and 87.7 ± 5.6% healthy roots in three different Taiwanese provenances of *Q. variabilis* (Jung et al., 2014).

The aim of this study is to assess the proteomic and metabolomic changes that the roots and shoots of *Q. suber* and *Q. variabilis* undergo during the first 36 h of *P. cinnamomi* infection. This approach can provide information about differences in how plants adjust the balance between primary and secondary metabolism, and about which defense mechanism are activated at each stage of the early infection (Saiz-Fernández et al., 2020). By comparing two tree species of different origins, this work tries to find the main molecular mechanisms responsible for the differences in susceptibility. In order to study the defense responses of trees, however, it is necessary to perform detailed and properly replicated experiments (Fenning, 2019); but, because of the high intraspecific genetic variability found in the *Quercus* genus (Coelho et al., 2006b; Carvalho et al., 2009), each plant within the same species can display a slightly different defense response. Therefore, this study will use micropropagated clonal plants to minimize this intraspecific variability by providing uniform and healthy plant material (Cahill et al., 1993; Fossdal et al., 2012; Nelson et al., 2014).

## Materials and Methods

### Plant Material

Acorns from the selected mother trees of *Q. variabilis* were collected in a natural mountain forest in Taiwan in November 2016, while acorns of *Q. suber* were obtained from Faro, Portugal in October 2017. Only healthy acorns without apparent damages caused by insects were selected. Ten acorns of both species were pre-germinated on wet tissue paper for 1 week in the dark. Germinated acorns were sown in autoclaved mixture of peat, vermiculite, and sand (1:1:1 v:v:v), placed in the growth chamber with 23/20°C (day/night) temperature and 8 h/16 h (day/night) photoperiod.

For each *Quercus species*, a single clonal line, showing the highest rooting rates, was selected for infestation experiments. Apical and nodal segments from a single 3-months old seedling of each *Q. variabilis* and *Q. suber* were used as initial explants for all further micropropagation. Explants were washed for 10 min with few drops of commercial detergent under running tap water. In the laminar flow cabinet explants were surface sterilized in a solution of 0.5% sodium hypochlorite (chlorine 11%) plus a few drops of Tween 20, agitating for 15 min followed by three rinses in sterile distilled water. Isolated explants were treated in 100 mg/L solution of ascorbic acid for 30 min to prevent oxidation. Shoot multiplication was achieved by subculturing explants on Woody Plant Medium (WPM) 4 (McCown and Lloyd, 1981) (Duchefa Biochemie, Netherlands), supplemented with 3% sucrose, 0.7% agar, 0.2 mg/L N^6^-benzyladenine (BA). During multiplication stage, explants of *Q. suber* and *Q. variabilis* were subcultured every 2 and 3 weeks, respectively. Rooting of the newly formed shoots was achieved on half-strength WPM medium supplemented with 2% sucrose, 0.7% agar, 1 g/L of activated charcoal (only for *Q. suber*) and 5 mg/L indole-3-butyric acid (IBA) and after 7 days shoots were transferred on plant growth regulator free medium. pH of the multiplication and rooting media was adjusted to 5.6 before sterilization by autoclaving at 121°C for 20 min. Explants were maintained in a cultivation room at 23 ± 1°C under a 16 h/8 h (day/night) photoperiod.

### *Phytophthora cinnamomi* Zoospore Production

*Phytophthora cinnamomi* (isolate TJ 349, isolated from soil of the rhizosphere of cork oak trees in declining stands in the Algarve region, southern Portugal) was selected from the Phytophthora Research Centre^1^ culture collection and developed on 10% semi-solid complete V8A agar media (prepared with 100 mL/L of clarified V8 juice (Pfanner, Lauterach, Austria), 20 g/l of agar (Sigma Aldrich, St. Louis, MO, United States), 2 g/L of CaCO_3_, and 900 mL/L of distilled water), and incubated for 10 days at 20°C in the dark. Zoospores were produced according to the procedure previously published by Byrt and Grant (1979) with small modifications. Briefly, five *P. cinnamomi* agar plugs were taken from the edge of an actively growing colony and placed onto a Miracloth disc (Calbiochem, La Jolla, CA), (previously boiled three times and sterilized) on a fresh 10% V8 Agar plate for 15 days in the dark. The miracloth with mycelium were transferred aseptically to a 250 mL Erlenmeyer flask containing 100 mL of 5% clarified V8A liquid media. Cultures were incubated overnight (≈16 h) in an orbital shaker (100 rpm) at 20°C, under 3 × 30 W fluorescent lights to induce sporangial production. Liquid V8A media was decanted and the miracloth washed 3 times with 100 mL sterile mineral salt solution (MSS) [0.01M Ca(NO_3_)_2_.4H_2_O, 0.005M KNO_3_, and 0.004 M MgSO_4_.7H_2_O dissolved in 1 L distilled water and autoclaved at 121°C for 20 min]. MSS was supplemented with 1 mL 0.1 M C_10_H_12_N_2_NaFeO_8_ solution, previously sterilized by filtration through a 0.22 μm filter (Millipore). The miracloth culture with 100 mL MSS was incubated overnight, in the same conditions as previously explained. After 24 h, the solution was decanted and the miracloth was washed with 50 mL MSS, moved to a Petri dish and covered with 20 mL MSS. To induce synchronous cleavage and release of zoospores, the Petri dish was placed at 4°C in the fridge for 1 h and afterward it remained for 1 h at 20°C. Sporangial production and zoospore release was confirmed by viewing each plate under a light microscope.

Micropropagated *Q. variabilis* and *Q. suber* plants that had been growing on nutritional media for 6 months were collected carefully (to avoid mechanical damages) and their roots were thoroughly washed in running lukewarm water from remaining nutritional agar medium. Inoculation of plants was performed by submersing the roots in 5 mL MSS with ca 10^4^ zoospores/mL suspension, prepared fresh for each experiment using *P. cinnamomi* isolate TJ349. Inoculation was performed at sunrise, and plants were kept in the same photoperiod and temperature regime as they were grown throughout the 5 timepoints planned for analysis: 4, 10, 16, 24, and 36 hours after inoculation (hpi). Per experiment, a minimum of 50 plants were inoculated, and the same number of non-inoculated plants submersed in MSS were used as a control. At each time point, plant material (leaves and roots) from a minimum of 10 plants per treatment was collected, pooled together, flash frozen in liquid nitrogen, and stored at −80°C. All experiments were repeated four times. The final sample count was 2 oak species × 5 time points × 2 treatments × 2 tissues × 4 replicates = 160.

### Sample Extraction

Leaf and root samples were extracted according to the method by Salem et al. (2020). Briefly, homogenized samples (four biological replicates of 50 mg per treatment) were extracted by incubation with 1 ml of pre-cooled (+4°C) MTBE:MeOH (3:1, v:v) mixture, spiked with 50 μg of valine C13 per sample. The extracts were vortex and incubated for 30 min on the ThermoMixer at 4°C, 1,500 rpm. The samples were centrifuged for 10 min at 12,000 rpm at 4°C. The supernatant was collected, and the pellet was washed with 0.5 ml of methanol. After centrifugation (10 min, 12,000 rpm, 4°C), the supernatant was collected and combined with previous one and, after vortexing, 600 μL were aliquoted. 150 μL MTBE and 450 μL H_2_O were added to this aliquot, which then was vortexed on RETCH mill (60 s, 30 Hz) and centrifuged for 10 min at 12,000 rpm at 4°C. The polar phase, which contained primary and secondary metabolites, was stored at −80°C until being used for metabolic profiling by GC/MS. The pellet obtained in the extraction of metabolites was used for shotgun proteomic analysis by LC/MS.

### Shotgun Proteomics

The protein-containing pellet was dried in a lyophilizer and resuspended in 0.3 mL of buffer consisting of 8M urea and 100 mM AMBIC. Samples were incubated overnight at room temperature. Afterward, aliquots corresponding to 100 μg of protein were reduced, alkylated with iodoacetamide, digested with trypsin (1 μg per sample, Promega) and desalted by C18 SPE as described by Hallmark et al. (2020). Proteomic profiling was performed as described by Berka et al. (2020b). Aliquots of 2.5 μg of peptide were analyzed by nanoflow C18 reverse-phase liquid chromatography using a 15 cm column (Zorbax, Agilent), a Dionex Ultimate 3000 RSLC nano-UPLC system (Thermo Fisher Scientific, Waltham, MA, United States) and the Orbitrap Fusion Lumos Tribrid Mass Spectrometer (Thermo Fisher Scientific) as described by Hallmark et al. (2020). The measured spectra were recalibrated and compared against the *Q. suber* reference protein database (GCF_002906115.1_CorkOak1.0, 10.1038/sdata.2018.6) and against the *P. cinnamomi* protein database (JGI Portal—*Phytophthora cinnamomi* var *cinnamomi* v1.0: Project: 1003774, Phyci1_all_proteins_20120612) by Proteome Discoverer 2.4, using complementary search algorithms Sequest HT (Thermo Fisher Scientific) and MS Amanda 2.0 (Dorfer et al., 2014) with the following parameters: Enzyme—trypsin, max two missed cleavage sites; modifications—carbamidomethyl (Cys) and up to three dynamic modifications including met oxidation, Asn/Gln deamidation, N-terminal acetylation; MS1 tolerance—5 ppm (MS Amanda), 10 ppm (Sequest), MS2 tolerance—0.02 Da (MS Amanda), 0.1 Da (Sequest). Since there is no annotated proteome available for *Q. variabilis*, the proteomes of both species were searched against the *Q suber* proteomic database. Only proteins with at least two unique peptides were considered for quantitative analysis. The quantitative differences were determined by Minora (Horn et al., 2016), using precursor ion quantification followed by normalization to total peptide amount and background-based *t*-test. Protein abundances were calculated as the summed intensities of the top 3 peptides. In total, 193 samples were analyzed (counting technical replicates), representing at least four biological replicates. Reproducibility was evaluated by PCA and 13 outliers were removed from the dataset. The mass spectrometry proteomics data have been deposited to the ProteomeXchange Consortium via the PRIDE (10.1093/nar/gkab1038) partner repository with the dataset identifier PXD033162.

The amount of *P. cinnamomi* proteins present in the roots of inoculated plants was estimated by querying the measured peptide spectra against the *P. cinnamomi* proteome and filtering out those proteins found in uninoculated tissues, as they probably originated from evolutionarily conserved sequences shared between *P. cinnamomi* and oak trees. The summed abundances of the remaining 57 proteins were used to estimate the amount of pathogen present in the tissue. Statistical differences between treatments and time points were visualized according to Kruskal-Wallis test (non-parametric; *p*-value threshold = 0.05). Peptide-based detection of *Phytophthora* has a high sensitivity comparable to that of qPCR (Berka et al., 2020a), which is a method often used to estimate pathogen load in infected tissues (Catal et al., 2013; Ali et al., 2017; Evangelisti et al., 2017). Peptide-based quantification has shown good correlation with qPCR quantification of *Phytophthora* content, and allows the discrimination between species with different degree of susceptibility to *Phytophthora* within the same genus (Dufková et al., 2021). Thus, *Phytophthora cinnamomi* protein content estimation was used as a proxy for susceptibility level in *Quercus* species.

### Metabolite Profiling

An aliquot of 30 μL of the metabolite-containing polar phase was dried in a speedvac. Samples were then derivatized by 20 μL of methoximation solution (40 mg methoxyamine hydrochloride in 1 mL pyridine) and incubated for 90 min at 30°C with continuous shaking. After the incubation, 80 μL of the silylation solution [N-methyl-N-(trimethylsilyl) trifluoroacetamide] was added and the mixture was incubated for 30 min at 37°C with continuous shaking. Derivatized samples were measured using a Q Exactive GC Orbitrap GC-tandem mass spectrometer and Trace 1,300 Gas chromatograph (Thermo Fisher Scientific, Waltham, MA, United States) as described by Saiz-Fernández et al. (2020). Samples were injected using the split mode (inlet temperature 250°C, splitless time 0.8 min, purge flow 5.0 mL/min, split flow 6.0 mL/min) onto a TG-5SILMS GC Column (Thermo Fisher Scientific, 30 m × 0.25 mm × 0.25 μm) with helium as a carrier gas at a constant flow of 1.2 mL/min. Metabolites were separated with a 28 min gradient (70°C for 5 min followed by 9°C per min gradient to 320°C and finally 10 min hold time) and ionized using the electron ionization mode (electron energy 70 eV, emission current 50 μA, transfer line and ion source temperature 250°C). The MS operated in the full scan mode, 60,000 resolution, scan range 50–750 m/z, automatic maximum allowed injection time with automatic gain control set to 1e6, and lock mass (m/z): 207.0323. Data were analyzed by TraceFinder 4.1 with Deconvolution Plugin 1.4 (Thermo) and searched against the NIST2014, GC-Orbitrap Metabolomics library, and in-house library. Only metabolites fulfilling identification criteria (score ≥75 and ΔRI <2%) were included in the final list. The quantitative differences were determined by manual peak assignment in Skyline 20.1 (Pino et al., 2020), using the extracted ion chromatogram (2 ppm tolerance). The mass spectrometry metabolomic data have been deposited to MetaboLights with the dataset identifier MTBLS4687.

### Functional Annotation and Bioinformatics Analysis

The identified protein sequences were queried against the SUBA^2^ and NCBI^3^ databases to generate a list of *Arabidopsis thaliana* and *Quercus suber* homologs, respectively. Putative orthologs with the highest score were selected for downstream data analysis and interpretation. The Uniprot database^4^ was used to assign each of the putative Arabidopsis homologs to functional categories. A functionally annotated biological process network was constructed using the gene ontology (GO) enrichment analysis of the Cytoscape ClueGO plugin (Bindea et al., 2009), setting a Kappa score of 0.4 and a minimum *p*-value of 0.1, according to the two-sided hypergeometric test with Bonferroni pV correction. Network interaction analysis was done using the STRING database.^5^ Functional enrichment and pathway topology analysis was performed using the “Joint Pathway Analysis” tool implemented in MetaboAnalyst 4.0 (Chong et al., 2019). Partial least squares-discriminant analysis (PLS-DA) and the corresponding Variable Importance in Projection analysis (VIP) and correlation coefficients [p(corr)] were performed using SIMCA-P + software package (Version 14.0; Umetrics, Umea, Sweden) to integrate the abundance of significantly affected proteins in *Quercus* roots with the estimated amount of *Phytophthora* proteins found in that tissue. VIP values summarize the importance of each variable for the PLS-DA model and plotting VIP vs. p(corr) values enabled highlighting important variables in the model.

## Results

### Estimation of *Phytophthora cinnamomi* Protein Content Suggests Higher Amounts of the Pathogen in *Quercus suber* Than in *Quercus variabilis*

**FIGURE 1:**
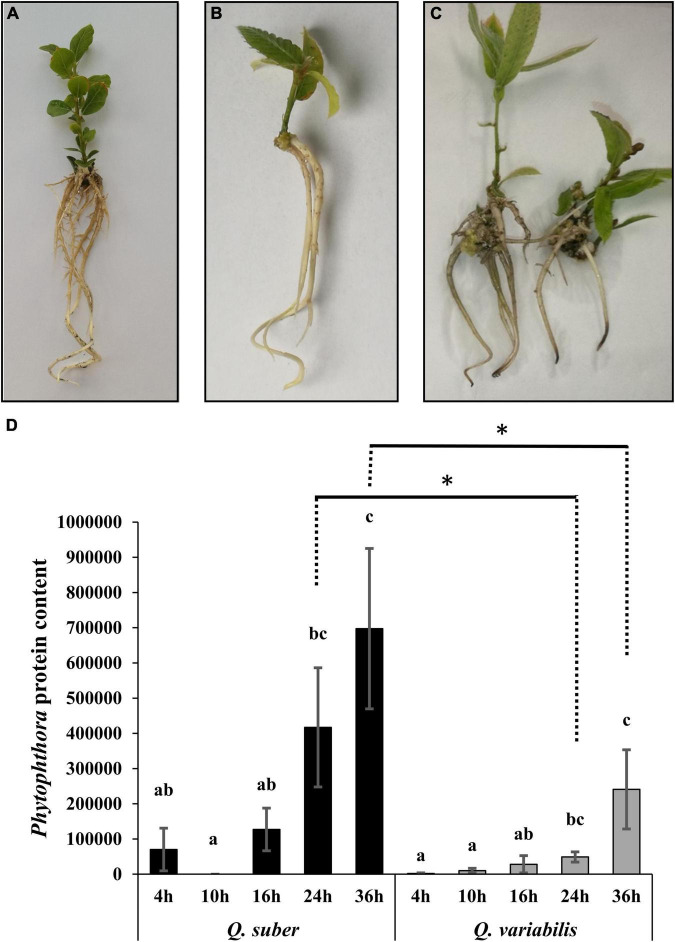
Visual status of micropropagated *Quercus suber* **(A)** and *Quercus variabilis* **(B)** seedlings prior to the inoculation, and of *Quercus variabilis* 36 h after infection with *Phytophthora cinnamomi* **(C)**. Total *Phytophthora*-origin peptide content found in each root sample **(D)**. Different letters indicate statistically significant differences between time points within each species according to Kruskal–Wallis test (*p* < 0.05). Asterisks indicate statistical differences between species for a given time point according to Kruskal–Wallis test (*p* < 0.05).

Micropropagated *Quercus* seedlings (Figures 1A,B) of both species displayed necrotic lesions in their roots 36 h after direct application of *P. cinnamomi* zoospore mixture (Figure 1C), which is a clear indicator of active *Phytophthora* infections in the tissue. The total amount of estimated *P. cinnamomi* proteins showed a time-dependent increase in the roots of inoculated plants, confirming the presence of the pathogen and indicating the advance of the infection (Figure 1D). In addition, the increase in *Phytophthora* protein content was greater in *Q. suber* plants, even though the differences were statistically significant only 24 and 36 hpi.

### The Difference in the Number of Affected Proteins Between Species Was the Highest 10 and 16 h After Inoculation

**FIGURE 2:**
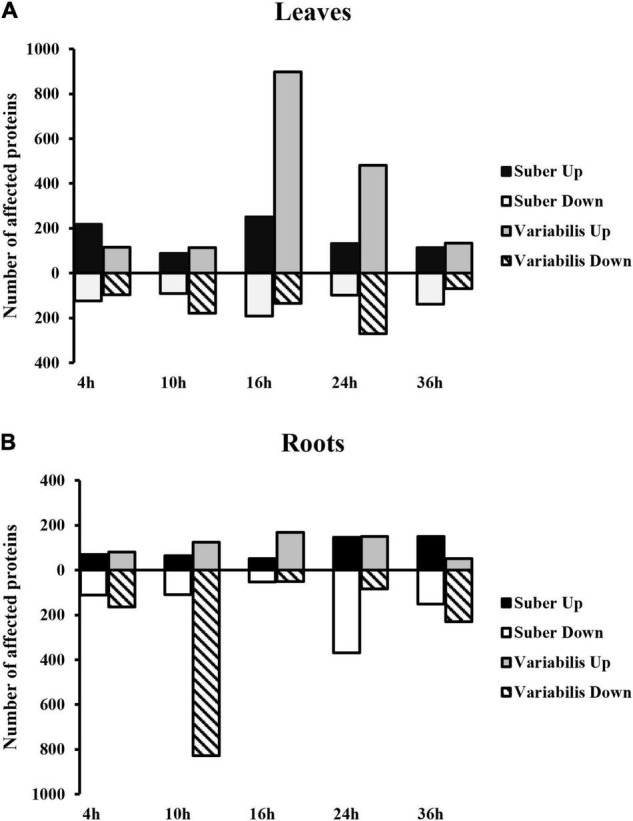
Number of proteins with affected abundance in the leaves **(A)** and roots **(B)** of *Q. suber* and *variabilis* plants after inoculation with *P. cinnamomi.*

**FIGURE 3:**
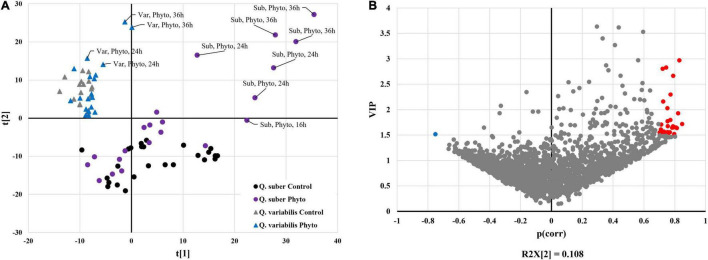
Partial least square discriminant analysis (PLS-DA) loading plot **(A)** and scatter plot p(corr)/VIP **(B)** based on the abundance of every significantly affected *Quercus* root protein and the total amount of *Phytophthora* proteins found in the roots of *Q. suber* and *Q. variabilis* plants. Highlighted proteins have a VIP score > 1.5 and a p(corr) > 0.7 (Red) or a p(corr) < −0.7 (Blue).

A total of 4,501 *Quercus* proteins were identified, out of which 1,434 and 2,355 proteins with 2 or more unique peptides were affected at some point of the infection process in *Q. suber* and *Q. variabilis*, respectively (Supplementary Table 1). The most pronounced differences in the number of affected proteins between species occurred at 10 and 16 hpi in roots and leaves, respectively (Figure 2). The PLS-DA integrating the abundances of *Quercus* root proteins with the estimated *Phytophthora* protein amount showed clear separation between the controls of both *Quercus* species (Figure 3A). However, only the samples with the highest *Phytophthora* protein content separated from their respective controls. This separation was more apparent in the case of *Q. suber*. Interestingly, despite the rater large number of proteins affected by *Phytophthora* inoculation, there was not much overlap between sampling points, regarding individual proteins, in neither of the two species. Similarly, when the root protein abundances were compared to the estimated amount of *Phytophthora* proteins a fairly low number of proteins showed a correlation and VIP scores above the required threshold (Figure 3B). Among the proteins positively correlated with *Phytophthora* content were a Universal stress protein (XP_023880172.1), an Endoglucanase (XP_023885388.1), an Annexin (XP_023889639.1), and a 3-Ketoacyl-CoA thiolase (XP_023922980.1); while the Elongation factor TuB (XP_023900391.1) was found to be negatively correlated with *Phytophthora* content (Supplementary Table 2).

### *Quercus variabilis* Displayed a Stronger Defense Response in the Leaves During the Initial 16 h of Infection

**FIGURE 4:**
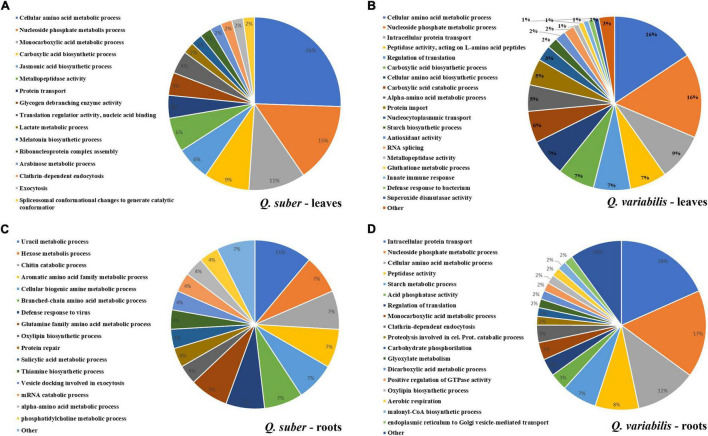
Functional annotation enrichment of proteins that were affected in the leaves **(A,B)** and roots **(C,D)** of *Q. suber* **(A,C)** and *Q. variabilis* **(B,D)** between 4 and 16 h after inoculation with *P. cinnamomi*. Pie charts depict functional categories assigned by the Cytoscape ClueGO plug-in using Arabidopsis orthologs of *Q. suber* proteins.

**FIGURE 5:**
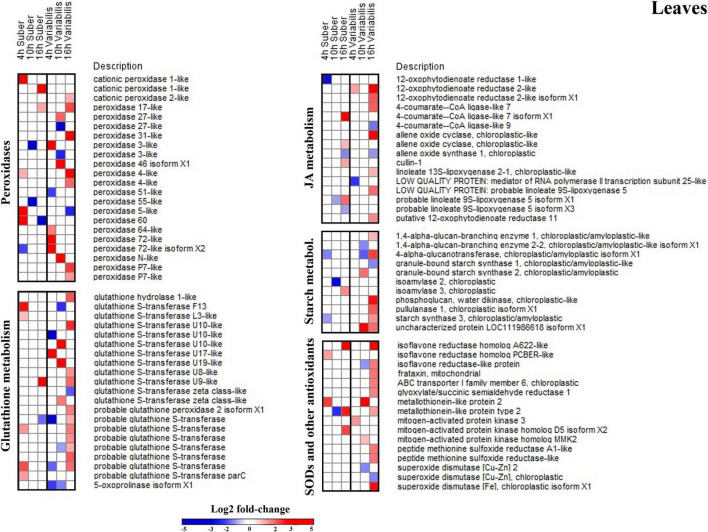
Log2-fold changes (|FC| ≥ 2, *p* < 0.05) in the abundance of leaf proteins related to several relevant biological processes in *Q. suber* and *Q. variabilis* 4, 10, and 16 h after inoculation with *P. cinnamomi*.

Functional annotation enrichment of leaf proteins affected during the first 16 h of inoculation revealed certain degree of overlap between the biological processes affected in both species (Figure 4 and Supplementary Tables 3, 4). Namely, amino acid, nucleoside phosphate, and carboxylic acid metabolism appeared overrepresented in both species. However, the extent to which these processes were affected at each time of the early infection process seems to be different between *Q. suber* and *Q. variabilis*. For example, in the case of *Q. suber*, the amino acid metabolism was predominantly affected 4 hpi, while in *Q. variabilis* 10 and 16 hpi registered a bigger number of regulated proteins (Figure 4C). In the case of carboxylic acid metabolism, no major upregulation of proteins was observed in neither species until 16 hpi, when both species showed an increase in TCA cycle-related protein abundances. However, this upregulation was more accentuated in *Q. variabilis* plants. Similarly, both species displayed increased abundance in proteins related to jasmonic acid metabolism and, despite this biological process only appearing as overrepresented in *Q. suber* plants (Figure 4A), the increase was also more pronounced in *Q. variabilis* plants (Figure 5).

Several biological processes related to plant defense response, such as glutathione metabolism or superoxide dismutase activity, were found to be overrepresented in the leaves of *Q. variabilis* plants only (Figure 4B). These processes appear to be strongly upregulated 16 hpi, as shown by the increase in the abundance of peroxidases, superoxide dismutases and other ROS-scavenging proteins, and glutathione metabolism-related proteins (Figure 5). These processes seem to also be upregulated in the leaves *Q. suber* plants, where this upregulation occurred earlier (4 hpi) but in a much lesser extent. Starch metabolism also appeared as overrepresented in *Q. variabilis* plants (Figure 4B) and, in fact, the abundance of several proteins related to this biological process increased 16 hpi (Figure 5).

**FIGURE 6:**
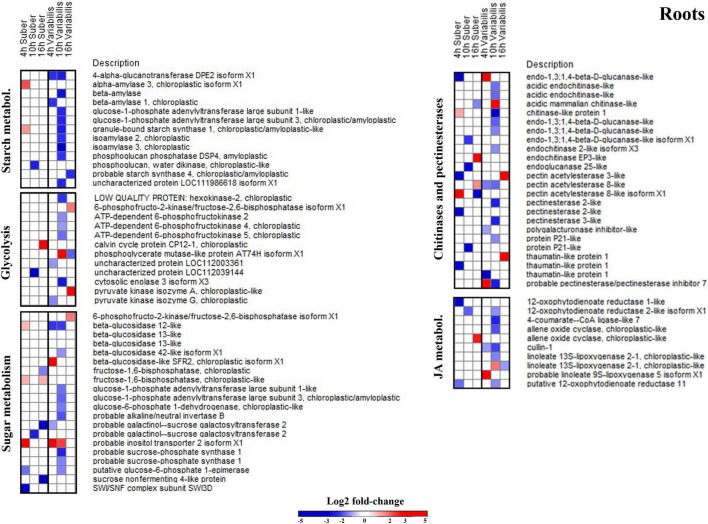
Log2-fold changes (| FC| ≥ 2, *p* < 0.05) in the abundance of root proteins related to several relevant biological processes in *Q. suber* and *Q. variabilis* 4, 10, and 16 h after inoculation with *P. cinnamomi*.

In the case of roots, the only biological process that appeared overrepresented in both species during the first hours after *P. cinnamomi* inoculation was oxylipin biosynthesis (Figures 4C,D and Supplementary Tables 5, 6), which is related to jasmonic acid metabolism; and indeed several proteins related to these metabolic pathways were affected in both *Q. suber* and *Q. variabilis*. Similarly to what was observed in the leaves, the number of affected proteins was higher in *Q. variabilis*, even though in this case the changes were the most pronounced 10 hpi. However, contrary to what was observed in the leaves, there was a decrease in the abundance of root proteins related to jasmonic acid and oxylipin biosynthesis (Figure 6).

Despite being overrepresented only in *Q. suber* (Figure 4C), the catabolic process of chitin and other cell wall macromolecules was affected in the roots of both species, as several chitinases, endochitinases, and pectinesterases were found to be affected after *P. cinnamomi* inoculation (Figure 6). The abundance of these proteins mainly decreased as a consequence of *Phytophthora* colonization, particularly in *Q. variabilis* 10 hpi. Contrarily, an increase in the abundance of several proteins of these families was observed in the leaves of *Q. variabilis* 16 hpi (Supplementary Table 1). The overrepresentation of hexose metabolism in *Q. suber* and pyruvate, carboxylic acid, and starch metabolism in *Q. variabilis* suggest that the carbon metabolism was affected, at least to certain extent, in both species (Figures 4C,D). To this regard, the abundance of root proteins related to starch and sugar metabolism, and glycolysis mainly decreased in *Q. variabilis*, particularly 10 hpi, while in *Q. suber* there was a relatively higher percentage of proteins with increased abundance (Figure 6).

### Both *Quercus* Species Showed Opposite Regulation of Shikimate Metabolism in the Leaves and Response to Sugars in the Roots 36 hpi

**FIGURE 7:**
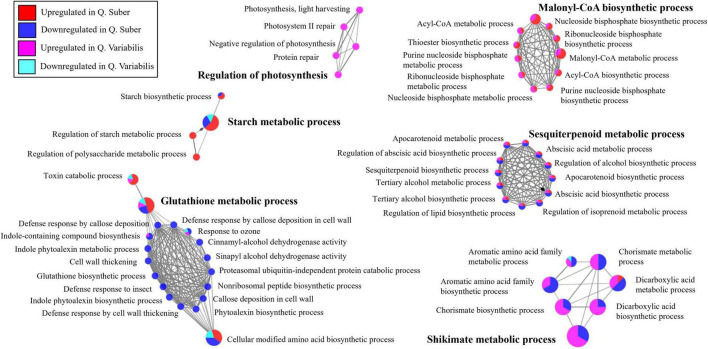
Functionally annotated biological process network constructed by ClueGO at kappa score ≥ 0.4 for leaf proteins affected in *Q. suber* and *Q. variabilis* 36 h after inoculation with *P. cinnamomi*.

The functionally annotated biological process network of *Quercus* leaf proteins shows how several biological processes related to defense response appeared to be downregulated in *Q. suber* 36 hpi (Figure 7). The exception to this was glutathione metabolism, which was upregulated in those plants. The shikimate/chorismite biosynthetic pathway, which is related to defense response, was also as downregulated in *Q. suber* plants. Conversely, this pathway appeared to be upregulated in the leaves of *Q. variabilis*. Similarly, the biological processes related to sesquiterpenoid metabolism also appeared to be oppositely regulated in both *Quercus* species, being mostly downregulated in *Q. suber* and upregulated in *Q. variabilis*. On the contrary, biological processes related to malonyl-CoA biosynthesis displayed a similar upregulation in both *Quercus* species. Oxylipin metabolism also showed upregulation in both species, albeit it was more apparent in *Q. suber*. Lastly, starch metabolism seemed to be upregulated in the leaves of *Q. suber* plants, only.

**FIGURE 8:**
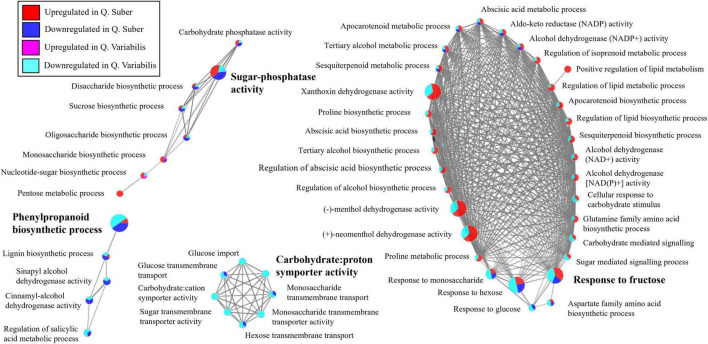
Functionally annotated biological process network constructed by ClueGO at kappa score ≥ 0.4 for root proteins affected in *Q. suber* and *Q. variabilis* 36 h after inoculation with *P. cinnamomi*.

In the case of the roots, the biological process network shows a downregulation of phenylpropanoid metabolism, including lignin biosynthesis, in both *Quercus* species 36 hpi (Figure 8). Similarly, carbohydrate transport also appeared as downregulated in both species, even though the effect was more dramatic in *Q. variabilis*. To this regard, processes related to the response to sugar levels and to the biosynthesis of di- and polysaccharides, including sucrose, were also downregulated in *Q. variabilis*. In the case of *Q. suber*, on the contrary, this downregulation was not that apparent, as these plants displayed both decreased and increased abundances of different proteins related to the aforementioned sugar-related biological processes. The differences between *Quercus* species were even more in the case of fatty acid catabolism, which was upregulated in *Q. suber*, but downregulated in *Q. variabilis*.

### There Was a Marked Decrease in Sugars in the Roots of *Quercus variabilis*

**FIGURE 9:**
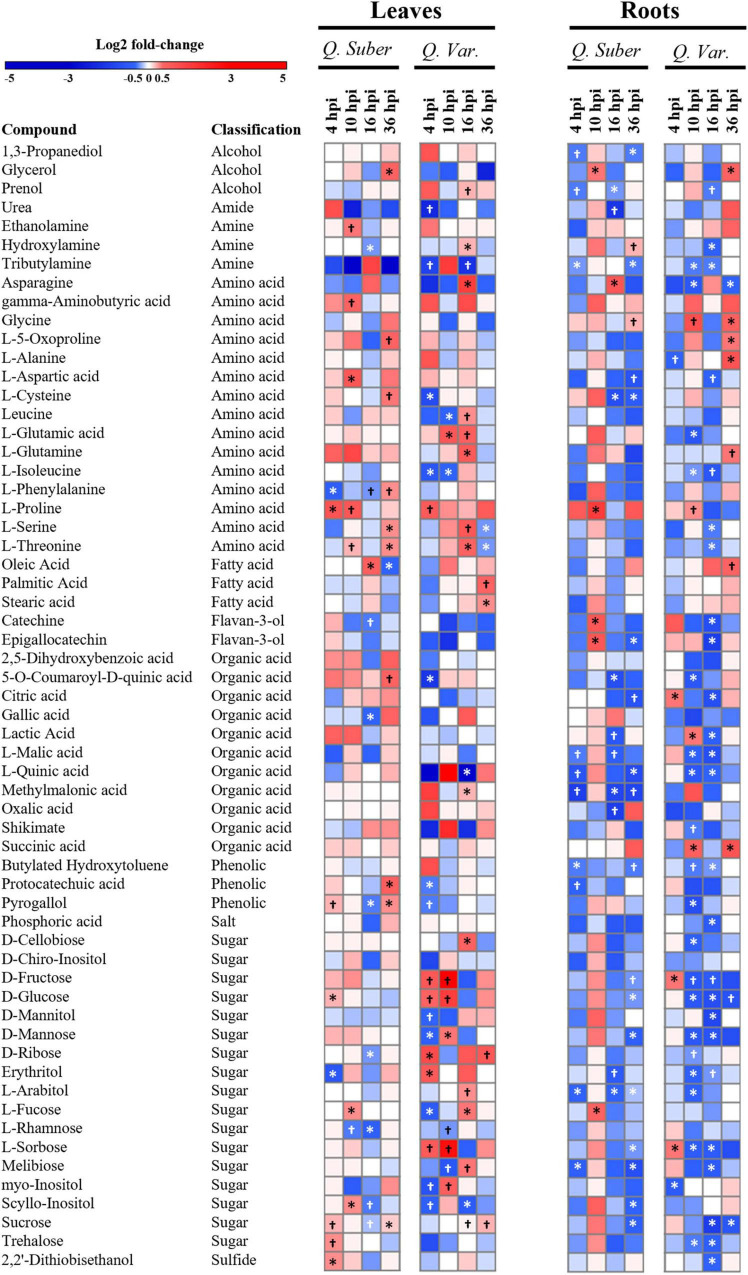
Heatmap showing log 2-fold changes in metabolites isolated from *Q. suber* and *Q. variabilis* leaves and roots after *P. cinnamomi* inoculation. Symbols indicate statistically significant differences according to Student’s *t*-test (^†^*p* < 0.1 and *p < 0.05).

In order to have a broader view of the effect that *P. cinnamomi* infection had on the main plant metabolic pathways, we analyzed the relative abundances of the most abundant metabolites (Figure 9 and Supplementary Table 7). In general, *Q. variabilis* showed a larger number of significantly affected metabolites (35 and 42 in leaves and roots, respectively) compared to *Q. suber* (26 and 32). However, despite the difference in the number of affected metabolites, both species displayed an overall decrease in the concentration of many roots metabolites as the infection progressed. This pattern was particularly striking in the case of certain sugars like D-glucose and D-fructose, whose concentration showed a sharp decrease in the roots of *Q. variabilis* 10 h post-inoculation. The concentration of sucrose, another of the main sugar forms, also decreased in *Q. variabilis* roots 16 hpi. In fact, there was a noticeable decrease in the concentration of many sugars in the roots of these plants 10 and 16 hpi. In the case of *Q. suber* roots, the decrease in the total content of identified sugars was most noticeable later in the infection process, 36 hpi; even though it occurred in less extent than in *Q. variabilis* (Supplementary Table 7).

Both species showed an increase of the stress-related amino acid L-Proline in the leaves during the first hours of infection, but the concentration remained high for a longer period in *Q. suber* plants (Figure 9). Proline concentration was also significantly increased in the roots of *Q. suber* plants 10 hpi, together with the Flavan-3-ols catechine and epigallocatechine. However, among these compounds only Proline increased in the roots of *Q. variabilis*, and to a lesser extent than in *Q. suber*.

**FIGURE 10:**
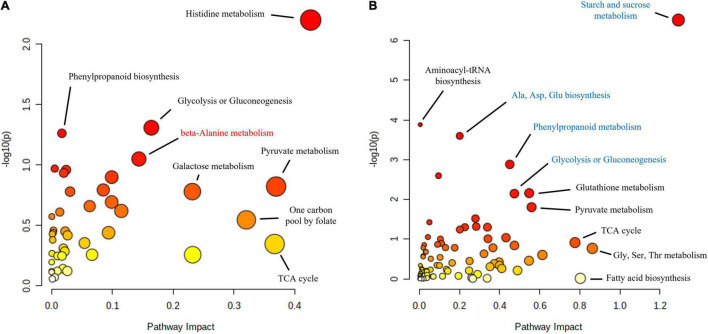
Functional enrichment and pathway topology analysis of the metabolites and proteins affected in the roots of *Q. suber* **(A)** and *Q. variabilis* **(B)** 10 h after inoculation with *P cinnamomi*. Molecular pathways highlighted in red and blue contained a higher number of proteins and metabolites with increased and decreased abundance, respectively. The color of each point corresponds to its *p*-value (yellow to red) and its size is calculated based on the pathway impact values.

**FIGURE 11:**
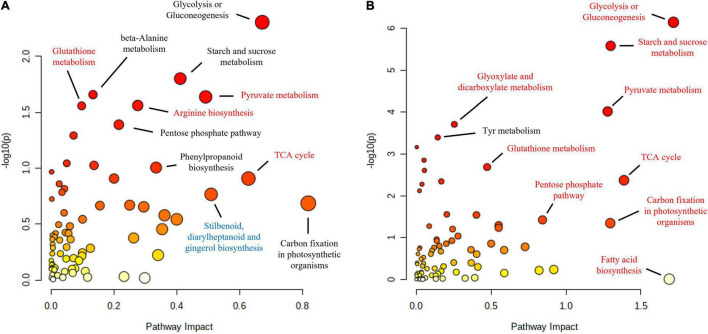
Functional enrichment and pathway topology analysis of the metabolites and proteins affected in the leaves of *Q. suber* **(A)** and *Q. variabilis* **(B)** 16 h after inoculation with *P cinnamomi*. Molecular pathways highlighted in red and blue contained a higher number of proteins and metabolites with increased and decreased abundance, respectively. The color of each point corresponds to its *p*-value (yellow to red) and its size is calculated based on the pathway impact values.

Functional enrichment analysis of the roots and shoots at the time when the maximum number of proteins were affected, 10 and 16 hpi in the roots and shoots, respectively, showed how the main affected pathways were related to carbon metabolism, particularly in the case of *Q. variabilis* (Figure 10). Starch and sucrose metabolism and glycolysis and gluconeogenesis were found to be repressed in the roots of *Q. variabilis* 10 hpi. On the contrary, these same pathways, together with pyruvate metabolism, TCA cycle, and pentose phosphate pathway, were found to be upregulated in the leaves of *Q. variabilis* 16 hpi (Figure 11). The upregulation of carbon metabolism was not as marked in the case of leaves of *Q. suber*, where only pyruvate metabolism and TCA cycle appeared as clearly upregulated. Glutathione metabolism, on the other hand, was found to be upregulated in the leaves of both species.

## Discussion

### Lignin and Phenylpropanoid Metabolism Are Part of the Early Defense Response in *Quercus variabilis* Roots

*Phytophthora cinnamomi* is one of the most aggressive tree pathogens in the world, and as such it is usually able to penetrate and colonize the roots of more than 5,000 host plant species, regardless of their level of susceptibility (Shearer et al., 2007; Allardyce et al., 2012; Hardham and Blackman, 2018; Camisón et al., 2019). Plant defense response in tolerant tree species tends to be based on stopping the progress of the pathogen within the host’s tissues and/or replacing the dead roots fast enough (Moreira and Martins, 2005; van den Berg et al., 2021). In agreement with these reports, within 36 hpi, the roots of both *Q. suber* and *Q. variabilis* developed necrotic tissue (Figure 1C). The estimation of *Phytophthora* content, based on the amount of unequivocally *Phytophthora* proteins found in the infected roots, further confirmed the presence of the pathogen in both *Quercus* species (Figure 1D). However, the *Phytophthora* protein content increased at a faster rate in *Q. suber* plants, suggesting faster colonization and a higher susceptibility of these plants to *P. cinnamomi*. This correlation between the variation in *Phytophthora* content, estimated either through qPCR-based or peptide-based approaches, and host susceptibility to the pathogen has been reported by previous studies (Vandemark and Barker, 2003; Catal et al., 2013; Dufková et al., 2021). The apparent difference in susceptibility between *Quercus* species might be attributed to the presumed area of origin of *P. cinnamomi* in south-east Asia (Jung et al., 2020; Shakya et al., 2021), which coincides with that of *Q. variabilis* (Wang et al., 2016) and might have allowed certain degree of co-evolution between host and pathogen (Jung et al., 2017, 2018, 2020). In fact, it has been reported how trees in native *Q, variabilis* forests in Taiwan appeared generally healthy, despite the presence of *P. cinnamomi* (Jung et al., 2017).

Early response against *Phytophthora* attack usually involve strengthening of cell walls, in order to stop or delay the penetration of the pathogen into the roots (Draper et al., 2018). This strengthening is done mainly through cell wall lignification processes, that are closely linked to phenylpropanoid metabolism (Vanholme et al., 2019; Saiz-Fernández et al., 2020). To this regard, both lignin and phenylpropanoid metabolism were overrepresented in the GO enrichment of proteins affected during the early response in *Q. variabilis* (Supplementary Table 2). More specifically, proteins like Laccase-14 (XP_023928547.1), Peroxidase 4-like protein (XP_023928860.1), probable Caffeoyl-CoA O-methyltransferase (XP_023898907.1), Leucoanthocyanidin dioxygenase (XP_023876832.1), and probable Cinnamyl alcohol dehydrogenase (CAD) 1 (XP_023912945.1) showed increased abundance in the roots of *Q. variabilis* 4 hpi with *P. cinnamomi* (Supplementary Table 1). In addition to contributing to the lignification process, phenylpropanoids have been linked to plant hypersensitive response, and exhibit a broad-spectrum antimicrobial activity (Draper et al., 2018). Moreover, CADs have been shown to inactivate *Phytophthora* aldehyde aromatic effectors by reducing them into alcohols (Coelho et al., 2006a). Flavonoids, which are synthesized via the phenylpropanoid pathway, are secondary metabolites which have also been linked to defense response to *Phytophthora cinnamomi* (Saiz-Fernández et al., 2020). Several proteins related to the biosynthesis of flavonoids and coumarins, such as Flavonoid 3’-monooxygenase (XP_023928106.1), Shikimate O-hydroxycinnamoyltransferase (XP_023913372.1), Transcription factor MYC2-like (XP_023900599.1), and several 4-Coumarate CoA ligases (XP_023914730.1, XP_023914151.1, XP_023911786.1), were also found to be upregulated in the leaves of *Quercus* seedlings (Supplementary Table 1), further suggesting the role of these compounds in plant defense against this pathogen.

### After the First Couple of Hours, Plant Defense Response Seems to Shift to Strategies Involving the Starvation of the Pathogen

Because some pathogens are capable of altering the transport of carbon and nitrogen resources between different plant tissues, the interpretation of metabolic data can be complex (Parker et al., 2009; Draper et al., 2018). The overall decrease in primary metabolites observed in the roots of *Q. suber* and, particularly, *Q. variabilis* (Figure 9) could be a consequence of the pathogen feeding on plant’s resources (Judelson et al., 2009; Corcobado et al., 2022) or the plant restricting the transport of photoassimilates toward the infected areas upon pathogen recognition (Tenenboim and Brotman, 2016; Corcobado et al., 2022). However, the decrease in sugars, including D-Glucose, D-Fructose, Sucrose, observed earlier in the roots of *Q. variabilis*, which showed a smaller amount of *Phytophthora* proteins (Figure 1B), suggests that their decrease was part of the plant defense mechanism. Moreover, the decrease in abundance of several proteins related to glycolysis and sugar and starch metabolism in the roots of *Q. variabilis* plants 10 hpi (Figures 6, 10B) further suggests that the decrease in sugar was a consequence of the plants’ actions. What is more, 36 hpi the metabolism of di- and polysaccharides remained downregulated in the roots of *Q. variabilis*, while sugar transport appeared downregulated in both species (Figure 8), highlighting the role of pathogen starvation as a long-term defense strategy against *P. cinnamomi*.

To this regard, it has been reported how during *Phytophthora* attack plants can downregulate their primary metabolism, shifting to a more defense-specialized secondary metabolism (Mansfeld et al., 2020). However, no such upregulation of defense response was observed in the roots of neither *Quercus* species, and the few identified secondary metabolites are not enough to asses such shift toward defense-specialized metabolic pathways. On the contrary, in the case of *Q. variabilis* a strong downregulation of proteins normally associated to pathogen defense, like endochitinases and pectinesterases, and to jasmonic acid metabolism was observed 10 hpi (Figure 6); proteins that have previously shown increased abundance in response to *Phytophthora* (Jalil et al., 2015; Xiao et al., 2019; Saiz-Fernández et al., 2020). Some authors have suggested that *Phytophthora*-originated elicitins can be responsible of the downregulation of these defense-related proteins (Oßwald et al., 2014). However, the fact that the downregulation was most noticeable in *Q. variabilis* plants, which had a lower *Phytophthora* content, suggests that there may be a different, yet unknown, reason for the decrease in the abundance of these defense-related proteins. Similarly, the reason for the increased abundance of several of these endochitinases and pectinesterases in the leaves of *Q. variabilis* 16 hpi is also unclear since these proteins require direct contact with the pathogen to be effective.

### Leaf Glutathione S-Transferases Could Play an Important Role in the Defense Response of *Quercus variabilis* Against *Phytophthora cinnamomi*

The increase in oxidative stress is a common response to pathogen attack in plants. This can occur either as part of plant defense mechanisms, since superoxide anion can be employed to directly harm the pathogen and trigger programmed cell death, and hydrogen peroxide can act as a signaling molecule to trigger lignification processes; or as a part of the pathogen attack strategy, since *Phytophthora*-triggered H_2_O_2_ can harm the host and facilitate colonization (Ge et al., 2013; Ebadzad et al., 2015; Hardoim et al., 2016; Judelson and Ah-Fong, 2019). In either case, the oxidative stress tends to be accompanied by increase in scavengers of reactive oxygen species (ROS), such as peroxidases, superoxide dismutases, and antioxidants like glutathione (Pandhair and Sekhon, 2006). However, the proteomic data did not reveal any meaningful increase in ROS-scavenging mechanisms in the roots of neither *Quercus* species. On the contrary, several of these proteins displayed increased abundance in the leaves of *Q. suber* 4 hpi and an even higher number of them showed upregulation in the leaves of *Q. variabilis* 16 hpi (Figure 5), which could be interpreted as a signal of abiotic stress (Choudhury et al., 2017). In fact, glutathione metabolism and superoxide dismutase activity were among the biological processes that appeared enriched in the leaves of *Q. variabilis* (Figure 4A), and glutathione metabolism was also highlighted in the functional enrichment analysis (Figure 11B). To this regard, glutathione S-transferases (GTS) in particular have been suggested to confer tolerance to various biotic stresses, including hemibiotrophic pathogens (Fortunato et al., 2015; Serrazina et al., 2015; Gullner et al., 2018). The abundance of several Aldehyde dehydrogenases (XP_023917826.1, XP_023903338.1, XP_023918652.1, and XP_023924290.1) was also increased in the leaves of *Quercus* seedlings (Supplementary Table 1). Aldehyde dehydrogenases have been linked to oxidative stress, as they can detoxify the ROS-produced aldehydes into carboxylic acids (San-Eufrasio et al., 2021). The upregulation of the most important pathways of C metabolism observed in the leaves 16 hpi, particularly in the case of *Q. variabilis* (Figure 11), could have been a way for the plants to boost the input of resources into these defense-related pathways of secondary metabolism. The up-regulation of C metabolism not only provides energy for plant secondary metabolism, but has also been suggested to positively regulate the expression of defense-related genes (Rojas et al., 2014). Unfortunately, the untargeted metabolomic analysis performed in the present study did not allow for the identification of these secondary metabolites, and a more exhaustive metabolomic study would be needed to confirm the shift of resources into these pathways.

Plant antioxidant machinery is usually induced in leaves, even in the case of infection by root pathogens, but these tend to be longer-term secondary effects caused by the infection (Moreno-Chacón et al., 2013), usually as a consequence of the *Phytophthora*-associated drought stress (Meyer et al., 2016). Here, on the other hand, the changes were observed only few hours after the infection process and are therefore unlikely to be drought-related. Interestingly, some studies have suggested that the upregulation of the antioxidant machinery can be beneficial for the pathogen in early phases of the infection, as it can disrupt plant oxidative burst-based defenses (Gullner et al., 2018). In fact, upregulation of GTSs is required for disease susceptibility against *P. parasitica* in tobacco plants (Hernández et al., 2009). This could explain the differences in the abundance of antioxidant-related proteins between both *Quercus* species (Figure 5), as the early increase in abundance observed in *Q. suber*, the more susceptible one, could be aiding the pathogen, while the more marked increase observed in the later stages in *Q. variabilis* could be helping the plant. This also highlights the extent of the differences in the defense response between timepoints only a few hours apart, as plants need to perform very quick adjustments in their metabolism to match different phases of infection, and therefore, individual proteins do not display a linear correlation with the infectious process.

In conclusion, there were marked differences in the proteomic and metabolomic response of both *Quercus* species during the first 36 h of *P. cinnamomi* infection. While both species showed a decrease in root sugars, it occurred earlier and in a greater extent in *Q. variabilis*; species in which a decrease in root proteins related to carbon metabolism was also observed. *Q. variabilis* also displayed a greater upregulation of stress-related proteins in the leaves, compared to *Q. suber*; namely peroxidases, superoxide dismutases, and glutathione S-transferases, together with proteins related to jasmonic acid metabolism. These differences in the defense response could have been responsible for the higher susceptibility of *Q. suber* to *P. cinnamomi* attack.

## Data Availability Statement

The original contributions presented in the study are included in the article/Supplementary Material, further inquiries can be directed to the corresponding author.

## Author Contributions

IS-F, BB, MČ, MH, and TJ: conceptualization. IS-F and MH: data curation. IS-F, BĐ, MB, MH, and PK: formal analysis. IS-F, BĐ, MČ, MH, PK, and TJ: methodology. TJ, BB, and MČ: funding acquisition. IS-F: writing—original draft. IS-F, BB, BĐ, C-HF, MB, MČ, MH, PK, and TJ: writing—review and editing. All authors have read and agreed to the published version of the manuscript.

## Conflict of Interest

The authors declare that the research was conducted in the absence of any commercial or financial relationships that could be construed as a potential conflict of interest.

## Publisher’s Note

All claims expressed in this article are solely those of the authors and do not necessarily represent those of their affiliated organizations, or those of the publisher, the editors and the reviewers. Any product that may be evaluated in this article, or claim that may be made by its manufacturer, is not guaranteed or endorsed by the publisher.

## Footnotes

1
www.phytophthora.org

2
https://suba.live/

3
https://www.ncbi.nlm.nih.gov

4
https://www.uniprot.org/

5
https://string-db.org/
